# The Development of Overtrust: An Empirical Simulation and Psychological Analysis in the Context of Human–Robot Interaction

**DOI:** 10.3389/frobt.2021.554578

**Published:** 2021-04-13

**Authors:** Daniel Ullrich, Andreas Butz, Sarah Diefenbach

**Affiliations:** ^1^ Department of Computer Science, LMU Munich, Munich, Germany; ^2^ Department of Psychology, LMU Munich, Munich, Germany

**Keywords:** human–robot interaction, overtrust, prior experience, reputation, demonstration, psychological perspective

## Abstract

With impressive developments in human–robot interaction it may seem that technology can do anything. Especially in the domain of social robots which suggest to be much more than programmed machines because of their anthropomorphic shape, people may overtrust the robot's actual capabilities and its reliability. This presents a serious problem, especially when personal well-being might be at stake. Hence, insights about the development and influencing factors of overtrust in robots may form an important basis for countermeasures and sensible design decisions. An empirical study [*N* = 110] explored the development of overtrust using the example of a pet feeding robot. A 2 × 2 experimental design and repeated measurements contrasted the effect of one's own experience, skill demonstration, and reputation through experience reports of others. The experiment was realized in a video environment where the participants had to imagine they were going on a four-week safari trip and leaving their beloved cat at home, making use of a pet feeding robot. Every day, the participants had to make a choice: go to a day safari without calling options (risk and reward) or make a boring car trip to another village to check if the feeding was successful and activate an emergency call if not (safe and no reward). In parallel to cases of overtrust in other domains (e.g., autopilot), the feeding robot performed flawlessly most of the time until in the fourth week; it performed faultily on three consecutive days, resulting in the cat's death if the participants had decided to go for the day safari on these days. As expected, with repeated positive experience about the robot's reliability on feeding the cat, trust levels rapidly increased and the number of control calls decreased. Compared to one's own experience, skill demonstration and reputation were largely neglected or only had a temporary effect. We integrate these findings in a conceptual model of (over)trust over time and connect these to related psychological concepts such as positivism, instant rewards, inappropriate generalization, wishful thinking, dissonance theory, and social concepts from human–human interaction. Limitations of the present study as well as implications for robot design and future research are discussed.

## Introduction

Today, it may seem that technology can do anything: from medical surgeries to cleaning jobs in our households, many tasks are nowadays performed by robots. Being faced with such impressive developments, people tend to overlook that technology which still has limits. Especially in the domain of social robots, which through their anthropomorphic shape may suggest to be much more than programmed machines, people may overtrust the robot's actual capabilities and reliability—and even explicit demonstrations of the robot's limits are not effective preventions. In a recent study (Robinette et al., 2017), an emergency evacuation scenario was simulated by spreading smoke and activating a fire alarm and an emergency evacuation robot was supposed to lead people to the nearest exit. Tragically, the participants followed the robot even when it performed faulty in a previous demonstration and even when they noticed that the robot was going in a wrong direction. Overtrust presents a serious problem, especially when it comes to sensitive domains in which lives or personal well-being might be at stake. On the other hand, besides overtrust, distrust could prevent effective human–robot interaction (HRI) as well. With distrust, human operators do not use but turn off or even consciously disable systems that can help them. Both types of miscalibrated trust represent severe problems, also for other applications of robots and intelligent systems such as automated stock trading systems (Folger, 2019), surgery robots (Clinic, 2019), or in general, robotic coworkers.

Another prominent example of miscalibrated trust is the automotive context and particularly autonomous driving, as discussed in relation to the recent series of accidents with Tesla cars. As reported, several drivers assumed to have a self-driving car instead of partial automation. They trusted that the system could do more than it was actually capable of and took their hands off the wheel in other situations than its limited, intended field of application (Giesen, 2016). Overall, the tendency to trust in technology beyond its actual capabilities seems widespread, and overtrust in an emergency evacuation robot or assisted driving are just two instances of a more general phenomenon.

The more innovative the domain, the more difficult it may be for people to assess the capabilities and limits of a technology. This makes the exploration of overtrust and possible countermeasures highly relevant for HRI and especially for the interaction with social robots, designed to evoke affect, emotion, and probably trust and acceptance. However, the relevant mechanisms may not be specific for the domain of robots but be related to general psychological effects and cognitive biases. Knowing what creates overtrust, in turn, may help to address this issue in the design and application of robots.

## Research Questions

Our research aims at a more profound understanding of the development of overtrust in the context of HRI and beyond. In particular, we are interested in the psychological mechanisms and biases that may foster the development of overtrust. As known from many situations of everyday life, a common problem is that people take their previous positive experience as a proof for their belief and trust in whatever seems convenient (e.g., Bye, 2012). For example, when arguing about whether it is safe to use unboiled tap water for preparing baby nutrition, a mother saying "I have raised four children and they all survived" might take this as a proof for trusting tap water, while it remains unclear whether she is right or just lucky. A similar effect could play a role in the domain of trust in technology. Instead of seeking potentially helpful external sources of information, and profiting from statistics and experiences of others, people often concentrate on what they assume plausible based on their personal prior experience. As long as their experience does not stand against it, people may readily trust a system without noticing that repeated positive experience does not imply actual reliability. Just because no accident has happened so far when taking one's hand off the wheel, this does not mean that the car is actually capable of fully managing the driving task in all situations—but people behave as if it could. Such an inappropriate usage of assisted driving systems may be interpreted as overtrust.

In parallel to such cases and as a working definition, we refer to overtrust as a phenomenon when a person seemingly trusts—or at least uses—a system beyond its actual capabilities (see next sections for a detailed discussion of the concept of overtrust in the research literature). In other words, we interpret a person's behavior as expressing trust, although we do not know to what degree this would be reflected in a person's explicit ratings of a system's trustworthiness. This is in parallel to many of our everyday interactions, where we behave in a certain way (e.g., buying something to eat at the bakery around the corner, taking a medicine, and taking the airplane) and thereby express trust toward a person or a system, without explicitly stating or reflecting on that fact. However, also additional factors besides trust may affect such observable behavior and we possibly could have endless academic discussion about whether a particular behavior is actually a sign of trust or just "mindless" behavior. For example, also habituation toward warning signs or sensory stimuli may play a role, such as "I have become used to the red warning light in my car," without actually reflecting on whether I can still trust that the car will perform as flawlessly as before. Therefore, our research considers a person's decision to use a system as a proxy for (over)trust. This, however, only represents a snapshot within a more complex interplay of additional influencing variables between the psychological concept of trust on the one hand and system usage on the other hand.

Based on these considerations, our research centers around two main questions related to the development of overtrust: First, we explore the assumed paradigm of overtrust and the expected primary effect of previous experience. Second, we discuss possible additional influencing and de-biasing factors such as skill demonstration, reputation, and experience reports of others.

We will start by giving an overview of related work in the field, then present a general paradigm of overtrust, discuss a case study on the development of overtrust toward a robot, and connect our findings to related psychological concepts such as positivism, inappropriate generalization, and dissonance theory. In this sequence, our case study serves as an abstraction of the general assumed mechanism behind overtrust and allows a systematic exploration of various possible influencing factors in contrast. In order to minimize possible biasing factors such as personal prior experience with the system under exploration, we deliberately decided on a rather unusual example of HRI, namely a pet feeding robot. At the same time, the example of trust in the pet feeding robot allowed us to create a scenario of (hypothetically) high personal relevance, that is, taking care of or risking harm to one's beloved pet. Our actual interest, however, was to understand the general mechanisms contributing to overtrust, which is of high relevance to various application domains of robots and intelligent systems, such as our daily working environment.

## Related Work

This section summarizes recent research and literature reviews (e.g., Bagheri and Jamieson, 2004) on trust in robots and intelligent systems as well as overtrust and its influencing factors.

### Definitions and Different Levels of Trust

A review of trust definitions in general (e.g., Rotter, 1967; Barber, 1983; Rempel et al., 1985; Luhmann, 2018) highlights the multidimensionality of the concept, each focusing on different aspects of people's everyday usage of trust. For example, Luhmann (2018) emphasizes the role of trust as a method for reducing social complexity, arguing that without trust, an individual would be overwhelmed by the necessary number of decisions and controls. The sociologist Barber (1983) defined trust as a mental attitude an agent maintains regarding its social environment. In his view, trust results from accumulated individual experiences in a social system. Other approaches emphasize the aspect of vulnerability (Moorman et al., 1993; Johns, 1996; Rousseau et al., 1998), namely a person who trusts another takes a risk by doing so. Accordingly, Lee and See (2004), p. 51) define trust as "the attitude that an agent will help achieve an individual's goals in a situation characterized by uncertainty and vulnerability." In the field of HRI, a prominent definition is that of Wagner (2009), specifying trust as "a belief, held by the trustor [i.e., the agent who trusts] that the trustee [i.e., the one who is being trusted] will act in a manner that mitigates the trustor's risk in a situation in which the trustor has put its outcomes at risk" (Wagner, 2009, p. 31).

Referring to different levels of trust, many researchers use the concept of calibration. Calibration describes to which extent a person's trust in a technology corresponds to the technology's actual capabilities (Muir, 1987; Lee and See, 2004). Depending on the calibration between trust and capabilities, three levels of trust can be differentiated: calibrated trust, distrust, and overtrust (Zeit-Online, 2016). Calibrated trust means that the level of trust matches the technology's capabilities. Distrust means that the level of trust falls short of the technology's capabilities. Consequently, people may not benefit from technical progress and/or take more risk than necessary. For example, in 1988, there were operators who did not want to trust automated controllers in paper mills and thus could not profit from their benefits (Zuboff, 1989). Similarly, distrust in robots, which are actually optimized and often more reliable than humans in particular domains of work, may lead to unnecessary losses and risks of human lives. Finally, overtrust means that a person's trust exceeds the system capabilities. In extreme cases, humans may trust a robot to perform a task that it was never designed to do and thereby risk a complete mission failure. For instance, pilots of an Airbus A320 relied so heavily on an autopilot that they eventually were not able to act manually and caused an airplane to crash (Sparaco, 1995). Overtrust can also lead to skill loss or loss of vigilance during monitoring tasks, as discussed in the context of automated cars and medical diagnosis systems (Carlson et al., 2014). Such excessive trust in "intelligent" technology can be seen as a more extreme version of automation bias, that is, the tendency of people to defer to automated technology when presented with conflicting information (Mosier et al., 1992; Wagner et al., 2018). In parallel to this, overtrust has also been defined as a state in which "people accept too much risk because they think that the entity which they trust lowers that risk" (Robinette et al., 2016, p. 105). Referring to the specific case of overtrust in robots, Wagner et al., 2018, p. 22 defined this as "a situation in which a person misunderstands the risk associated with an action because the person either underestimates the loss associated with a trust violation, underestimates the chance the robot will make such a mistake, or both."

### Examples of Overtrust in Robots and Intelligent Systems

One of the most prominent recent examples of overtrust was the accidents caused by Tesla's autopilot. Tesla is a company located in the United States which produces electric cars (Tesla, 2019a). The first version of Tesla's autopilot (Hardware 1, 2014–Oct 2016) is an advanced driving assistance system classified as a level 2 automated system by the National Highway Transportation Safety Administration (SAE) (SAE-International, 2018). According to Tesla, "it is designed as a hands-on experience to give drivers more confidence behind the wheel, increase their safety on the road, and make highway driving more enjoyable by reducing the driver's workload" (Tesla, 2019b). In level 2 (partial automation), one or more driver assistance systems of both steering and acceleration/deceleration are active, for example, cruise control and lane-centering. However, the driver must still always be ready to take control of the vehicle and perform the remaining aspects of the driving task (SAE-International, 2018).

In May 2016 in Florida, a Tesla S crashed into a truck which was turning at a crossing. The reason for this accident was probably that the cameras of the car did not recognize the white side of the trailer truck and could not distinguish it from the sky, thus it was considering it a street sign (Zeit-Online, 2016). In another Tesla crash in China, the driver crashed into a car, which was parking near the guardrail; he survived. The driver acknowledged that he was not concentrating on the traffic. Instead, he was assuming that his Tesla could identify dangers and react accordingly. Based on the promotion of Tesla cars, he assumed having bought a self-driving car instead of a car with partial automation. Other customers in China confirmed this statement as they reported of vendors taking their hands off the wheel to show what the car is capable of, suggesting a deceptive understanding of the technology (Giesen, 2016).

Hence, in the following public discussion, the main concerns were not about the performance of the system but about the users' inadequate expectations. The term "autopilot" could encourage drivers to assume that they do not need to monitor the vehicle. This was further reinforced by anecdote user stories such as the report of Reek (2015) on his first drive in a Tesla using autopilot. He stated that after a few minutes, he already felt accustomed to the technology. He also tested what happened when he took his hands off the wheel. Instead of warning the driver immediately to place his or her hands back on the wheel, nothing happened. The reports of Reek and many other drivers on YouTube illustrate how easy it is for people to develop trust in a system, finally leading to irresponsible use: Even though Tesla's autopilot was still in a test phase, people started posting videos on YouTube, playing games, or sleeping and ignoring the warnings form Tesla's autopilot to place hands back on the wheel (Autobild, 2018). More and more people seemed to trust the system and forgot that the car has not been fully autonomous (Süddeutsche-Zeitung, 2016). The drivers felt comfortable and demonstrated irrational behavior, such as driving hands-free in their cars and playing games (Day, 2016). In addition, the motive to seek rewards from the YouTube audience may have cast all hesitations aside. One video, featured by a German radio moderator, even shows how he takes his hands off the wheel and instructs the car to change to the right lane. This is seriously critical as Tesla's autopilot still does not recognize cars with a speed of 300 km/h but this speed is allowed (albeit not frequently found) on certain motorways in Germany (Reek, 2015).

From the statistical point of view, self-driving cars may trigger far less accidents than human drivers and provide a huge potential from many perspectives. Innovations in this field could change the car insurance industry by reducing accidents: a report from the audit firm KPMG predicts that accidents will drop by 80% by 2040 (Albright et al., 2015). Employees could gain productive hours during the day by working instead of driving during daily commutes. Hence, after the first car crash emerged, Tesla already clarified that this was the first crash after 200 million completed kilometers, compared to one deadly car crash after an average of 150 million kilometers if a human is driving (Autobild, 2018). All the more, it is tragic that even the few deadly accidents might have been prevented if drivers had formed adequate levels of trust instead of overtrust.

Similar examples of overtrust can also be found in the domain of robots. As noted above, Robinette et al. (2016) studied trust in emergency evacuation robots. In one of their recent studies in a real-world environment (Robinette et al., 2017), they first showed a demonstration of an emergency evacuation robot to the participants, which was supposed to lead them to the nearest exit. In 50% of the cases, the robot failed and in 50% it succeeded. Afterward, the actual emergency evacuation scenario was simulated by spreading smoke and activating a fire alarm. To Robinette et al.'s surprise, the participants followed the robot even when it performed faulty in the demonstration, and even when they actually noticed that the robot was going into the wrong direction. This was surprising for Robinette et al. as in their former studies in virtual environments, where no direct harm was present, people did not follow the faulty robot (Robinette et al., 2016). Possibly, feeling actual danger may still enhance the risk for overtrust: in a secure situation in which no direct harm can be done to the user, trust in a faulty robot is lower than in an emergency situation in which the user's health is dependent on the robot's behavior. From a socio-psychological point of view, higher trust in robots in especially risky situations may also reflect a form of responsibility shift and diffusion of responsibility. Diffusion of responsibility describes the phenomenon that a person is less likely to take responsibility for action or inaction when others are present^1^. If this other may also be a robot, an emergency robot may also appear as an opportunity to share blame and guilt for a potentially bad outcome in severe situations.

Besides dramatic consequences for the users themselves (e.g., getting hurt in an accident), overtrust also threatens the manufacturer's image. Even if the technology did perform well within the spectrum of situations, it was built for, usage in situations beyond the system's capabilities result in a negative experience, a dramatic drop in trust, and an "unfair" negative reputation. The same effect of inappropriate generalization that may lead to overtrust (if it is good in situation A it must be good in situation B) then leads to distrust (if it failed in B it is a failure in general). Thus, from an individual, societal, and economic perspective, neither overtrust nor distrust is desirable.

### Trust in Robots and Parallels to Other Domains of Trust

Regarding the development of trust in robots and intelligent systems, prior research in two domains may be particularly informative: trust in automation and trust in humans. To some degree, trust in humans, automation, and robots are based on similar fundamental characteristics such as reliability, predictability, and ability (Jian et al., 2000). Empirical studies showed that trust in robots is strongly correlated to trust in automation (Sheridan, 2002; Lee and See, 2004; Parasuraman et al., 2008; Chen et al., 2010), and definitions in the context of trust in automated systems are typically applicable to trust in robots as well (Lee and See, 2004; Hancock et al., 2011). Starting from the definition of automation as "the execution by a machine agent (usually a computer) of a function that was previously carried out by a human" (Parasuraman and Riley, 1997, p. 231), robots expand the field by perception and intelligence and other important factors (Feil-Seifer and Mataric, 2005; Yagoda and Gillan, 2012). In addition, and in contrast to most automated systems, robots often even look similar to humans or animals. Consequently, robots may trigger psychological mechanisms from social interaction between humans, suggesting that research on trust between humans may also play a role here. Objectively considered, the mutual dynamics of trust among humans and trust between humans and (humanlike) artifacts bear fundamental differences. If, for example, someone trusts me, I will have the feeling that I must not disappoint this person. An artifact, on the contrary, will not have those feelings (Coeckelbergh, 2012). Despite these basic differences, it cannot be ruled out that people still transfer behavioral patterns from human–human interaction to human–robot interaction. As repeatedly shown, humans recognize robots as social actors (Keijsers and Bartneck, 2018): Humans talk to robots as if they understood what is being said (Bartneck et al., 2007), feel sorry for them when they are being punished (Slater et al., 2006), and try to prevent robots from getting hurt (Darling, 2012).

Related to the discussion of robots and computers as social actors, as it has already started in the 90's (Nass et al., 1994; Lee and Nass, 2003), is the factor of anthropomorphism. This means the application of human characteristics (form and behavior) to artificial agents such as robots (Bartneck et al., 2009). It is based on the tendency of a human to treat objects with humanlike appearance more like a human. Thus, appearance and behavior of the robot may cause its perceived intelligence and interaction (gestures and moving eyes) with the human to be increased (Cathcart, 1997; Qiu and Benbasat, 2009; De Graaf and Allouch, 2013). Accordingly, multiple studies have revealed that a robot's appearance can affect user's expectation, perception, and evaluation of its behavior and capabilities (Kiesler and Goetz, 2002; Goetz et al., 2003; Robins et al., 2004; Syrdal et al., 2007). Building on such insights, avoiding features that may nudge users toward anthropomorphizing robots have already been suggested as a possible starting point to mitigate overtrust (Wagner et al., 2018).

### Influencing Factors of Trust in Robots

Previous studies on potential influencing factors of the development of trust in robots and intelligent systems included the impact of users' knowledge of the system's capabilities (Sanchez, 2006), the recency of errors by the system (Sanchez, 2006), the timing of a robot's apologies for failure (Robinette et al., 2015), the assumed degree of user influence on the robot (Ullman and Malle, 2016), the particular effect of social and emotional human–robot interactions (Lohani et al., 2016), and others. In a literature review on trust in the domain of robots (Hancock et al., 2011), one of the most dominant influencing factors turned out to be reputation in the sense of knowledge about the robot's reliance (Bagheri and Jamieson, 2004) or knowledge about the robot' past performance (Lee and See, 2004; Chen et al., 2010). In general, reputation is defined as the "overall quality or character as seen or judged by people in general" and "recognition by other people of some characteristic or ability" (Merriam-Webster-Dictionary, 2019). Another central influencing factor of trust is the robot's actual performance, which may be experienced through real time feedback about the robot's performance (Hoffman et al., 2009; Chen et al., 2010). In general, performance is defined as "the execution of an action" and "the fulfillment of a claim, promise, or request" (Merriam-Webster-Dictionary, 2019). Judgments about the robot's performance may be inferred from demonstration (e.g., 2017; Robinette et al., 2016) or peoples' prior and current personal experience with a robot. If they repeatedly experience that the robot performs well, they build up trust in the robot in general, manifesting in positive expectations about the robot's future performance.

Besides reputation, demonstration, and personal experience as the central influencing factors of trust in robots, another relevant factor may be the humanlike nature of robots. As discussed above, people experience robots as social actors and often apply behaviors from human–human interaction (Keijsers and Bartneck, 2018). While a social relationship and similarity to humans are no prerequisites for trusting technology, a social relationship (as promoted in the case of social robots and other intelligent systems entering a dialog with the user) may make it even easier to build up trust. Consequently, different levels of "socialness" may also affect trust in robots. For example, Martelaro et al. (2016) studied the effects of different robot personalities such as a "vulnerable" robot personality, revealing that participants had more trust and feelings of companionship with a vulnerable robot.

## General Paradigm of Overtrust

**FIGURE 1:**
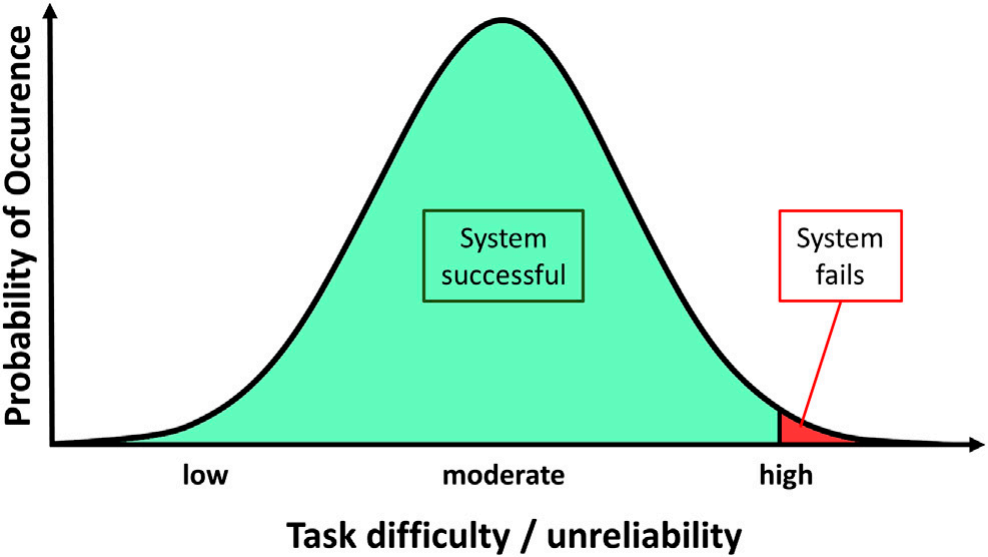
General paradigm of the development of overtrust.

We assume that the development of overtrust does not happen at random but follows specific inherent regularities in the interaction of system design, probability distribution, and human trust development. Thus, we suggest a general paradigm of the development of overtrust. Figure 1 illustrates this based on a hypothetical distribution, based on the following central considerations:

A technical system is designed to be capable to perform the tasks in the environment it is intended for. It is typically tested for these very situations with a certain degree of tolerance for both divergent tasks and environmental variables. It will fail, however, if the deviation between the intended and the actual application environment becomes too large.

Based on a standard distribution of probability, most crucial variables (e.g., task difficulty and disruptive factors) will spread around average values and will result in successful task accomplishment. System failure is a rare occurrence.

Every single interaction accumulates in the user's perceptions of the system and therefore results in a specific (change in the) degree of trust.

Since such a system is effective and successful in most cases, the participants will inevitably build up trust until it surpasses the level of calibrated trust, resulting in the development of overtrust. At this point, users will be more likely to use the system in inadequate situations (e.g., using an autopilot on a curvy mountain road) making system failures more probable.

Note that learning about the system's capabilities may not always be on an explicit level and one's ideas about what a system is capable of or not may not always be clear cut. In many cases, trust may be built on rather vague and intuitive associations based on unconscious, non-declarative memory systems, such as in the case of perceptual learning (e.g., Packard and Knowlton, 2002; Gazzaniga et al., 2006; Yin and Knowlton, 2006) and the improved abilities of sensory systems to respond to stimuli through repeated experience. Indeed, many of our everyday interactions rely on non-declarative learning, being typically hard to verbalize. For example, a mother that attends her child while walking along the street may predominantly rely on her intuitive feelings regarding the child's capabilities, based on her prior everyday experiences, without referencing specific developmental stages or declarative book knowledge about a child's cognitive abilities at a certain age. Depending on the mother's estimations to what degree the child is capable to realize danger, figure out the traffic situation, or can follow traffic rules, she may take the child by the hand or not. In the latter case, she (implicitly) trusts that the child will not perform any unexpected dangerous behavior such as suddenly running to the street. If this happens, nevertheless, that is, the child runs to the street although it never did before, this may also be denoted as a case of overtrust. Maybe, the mother overestimated the child's cognitive abilities. Maybe, the reason that the child did not run to the street before was that there never was a reason (e.g., seeing a friend on the other side of the street and a ball rolling to the street) and not that it realized that running to the street is dangerous. In parallel, users of the Tesla autopilot may have overestimated its abilities—but this discrepancy between expectations and actual capabilities behind a shown behavior did not become obvious until there was a critical situation which revealed the fatal misconception.

## Case Study: The Development of Overtrust in a Pet Feeding Robot

The following case study provides a simulation of the assumed general paradigm of overtrust using the example of a pet feeding robot. In the course of the study, the participants were presented with the hypothetical scenario of leaving their cat alone in their flat when going on holiday. To make sure the cat survives, they used a pet feeding robot. However, in their holiday location, they also had a possibility to check if the feeding was successful by means of a control call. In parallel to a risk and rewards perspective (e.g., driving hands off wheel to use the smartphone for entertainment), in our study scenario doing the control call was connected to missing another, possibly more entertaining option (i.e., a jungle trip). We assumed that depending on how much the participants trusted the robot, they would either make use of the control call or not. In order to explore the influencing factors of overtrust, the study design implemented a failure of the pet feeding robot after a certain number of successful feedings. Thus, those participants deciding against the control check in this scenario represented a case of "overtrust." One might critically question whether this type of overtrust is comparable to other cases since there are many differences to other contexts such as autonomous driving, AI medical decisions, or stock recommendations. However, the striking parallel is that for any reason, after a number of positive experiences, there may be cases when the system does not perform as previously experienced and trusting the system without critical questioning can have dramatic consequences.

Our study focused on three potential influencing factors of trust identified as central in prior research (see previous sections): one's personal experience with the robot, its reputation, and the demonstration of its capabilities. In addition, we checked the subjective relevance of such factors in a pre-study (sample size *N* = 186), where we presented a list of further potential influencing factors discussed in the literature (e.g., personality) and asked the participants to rate the most relevant factors for trusting a robot (1 = most important and 6 = least important). In parallel to previous literature reviews (Hancock et al., 2011), personal experience (M = 2.00), reputation (M = 3.35), and demonstration (M = 3.51) were rated the most important factors. In order to control for potential effects of personal involvement and emotional weight of the study scenario, we also surveyed whether participants actually owned a pet themselves and considered this as a control factor in statistical analyses. Also, we surveyed whether participants actually perceived the jungle trip as the more attractive option compared to the control call. If this was not the case, there was no obvious reason to miss the control call and put the system to the test, and therefore no basis to explore trust. In order to control for potential differences of the testing environment, the study was conducted as a lab study [subsample size *n* = 44] and an online study [subsample size *n* = 66]. One might assume that since the procedure contains annoying and boring parts, the participants in the online study might do other things alongside to make the study more enjoyable, which could bias the results. However, no significant differences were found between the two study environments. In the following sections, we thus present the pooled data of both study environments [sample size *N* = 110].

### Method

#### Participants and Study Procedure

The participants were recruited via mailing lists and incentivized by receiving course credit or amazon coupons. 110 participants took part in the study, 53.6% female, mainly students or people with academic background. The average age was 25.6 years (range 18–53, SD = 6.19). Personal experience with the robot was realized via repeated usage, in which the participants would collect experiences of the performance and reliability of the robot. Altogether, the scenarios consisted of 28 usage events. The influencing factors *capability*, *demonstration*, and *reputation* were experimentally manipulated, resulting in a 2 × 2 experimental design, consisting of two independent variables with two levels each.

Capability demonstration: The demonstration of the robot's capabilities was operationalized by means of a short video clip, showing a successful (positive) or faulty (negative) food preparation.

Reputation: The robot's reputation was realized via customers' reviews of the robot, containing enthusiastic (positive) or disappointed (negative) experiences.

**FIGURE 2:**
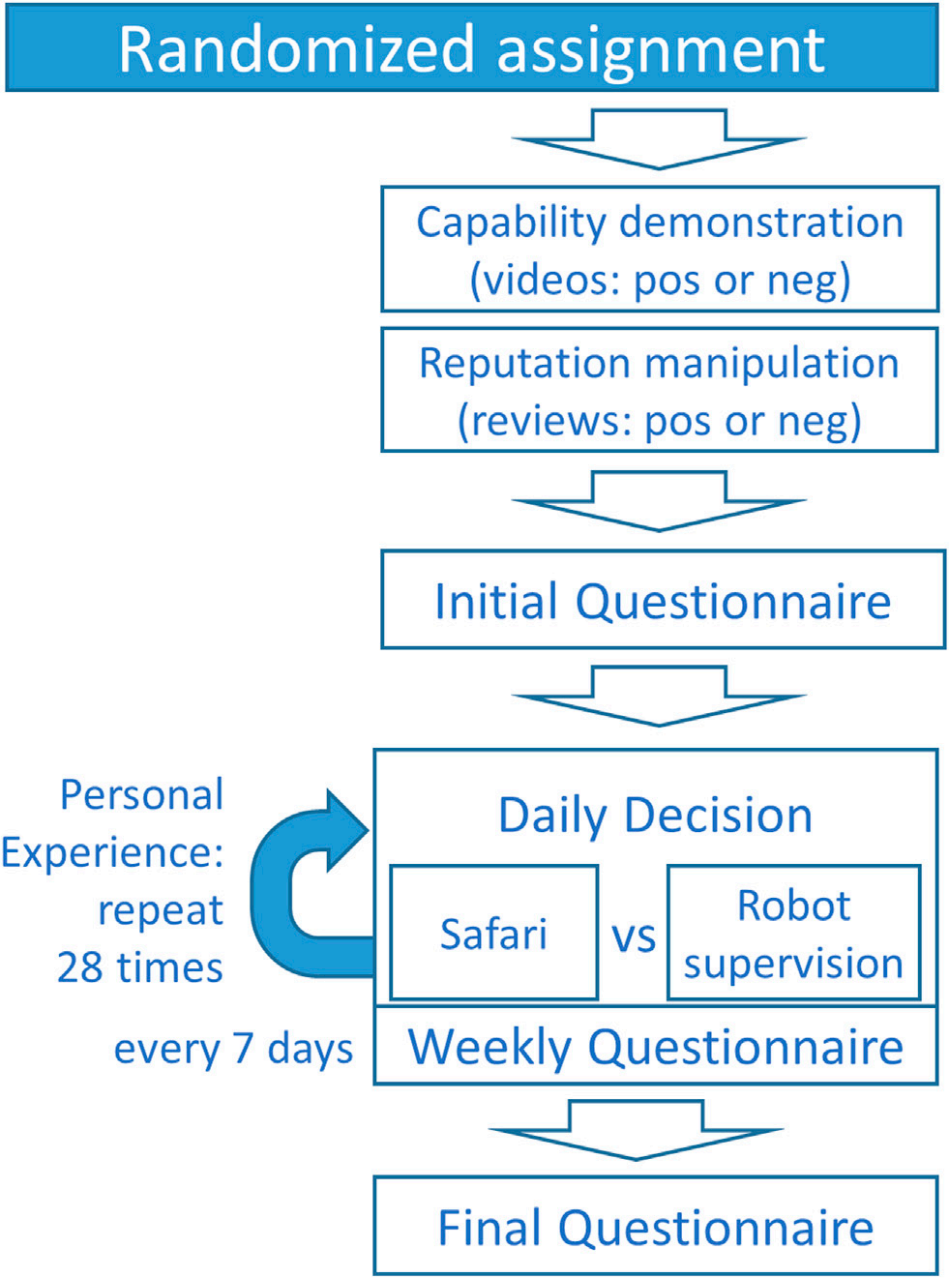
Study procedure.

The two factors were varied between subjects and the participants were randomly assigned to one of the four experimental groups. Figure 2 gives an overview of the study procedure and questionnaires.

The study scenario asked the participants to vividly imagine the following situation: "You are a tourist, going for a 28 days long safari trip and leave your beloved pet (a cat) at home. In order to ensure a regular feeding, you are using a pet feeding robot." In addition, the participants were told about the following context conditions:

1) The cat survives 2.5 days without feeding. This implies that after the second missed feeding, a call at your relatives (living in another town, who could do the feeding in case of emergency) should occur, or else the cat will die.
2) Every day, the participant has to make a choice: go to a day safari (having fun and learning interesting things about the jungle) or make a trip to another village to check if the feeding was successful (boring car trip).

In order to simulate the typical course of mainly positive experience with intelligent technology such as in the case of the Tesla autopilot, the feeding robot performed flawlessly most of the time. However, in the fourth week, it performed faultily on three consecutive days, resulting in the cat's death if the participants had decided to go on the day safari on these days. Note that this scenario (i.e., an unexpected technology performance, ending in a disaster, after a long period without realizing any problems or failures) was intentionally designed to create a ground for overtrust and its experimental investigation.

**FIGURE 3:**
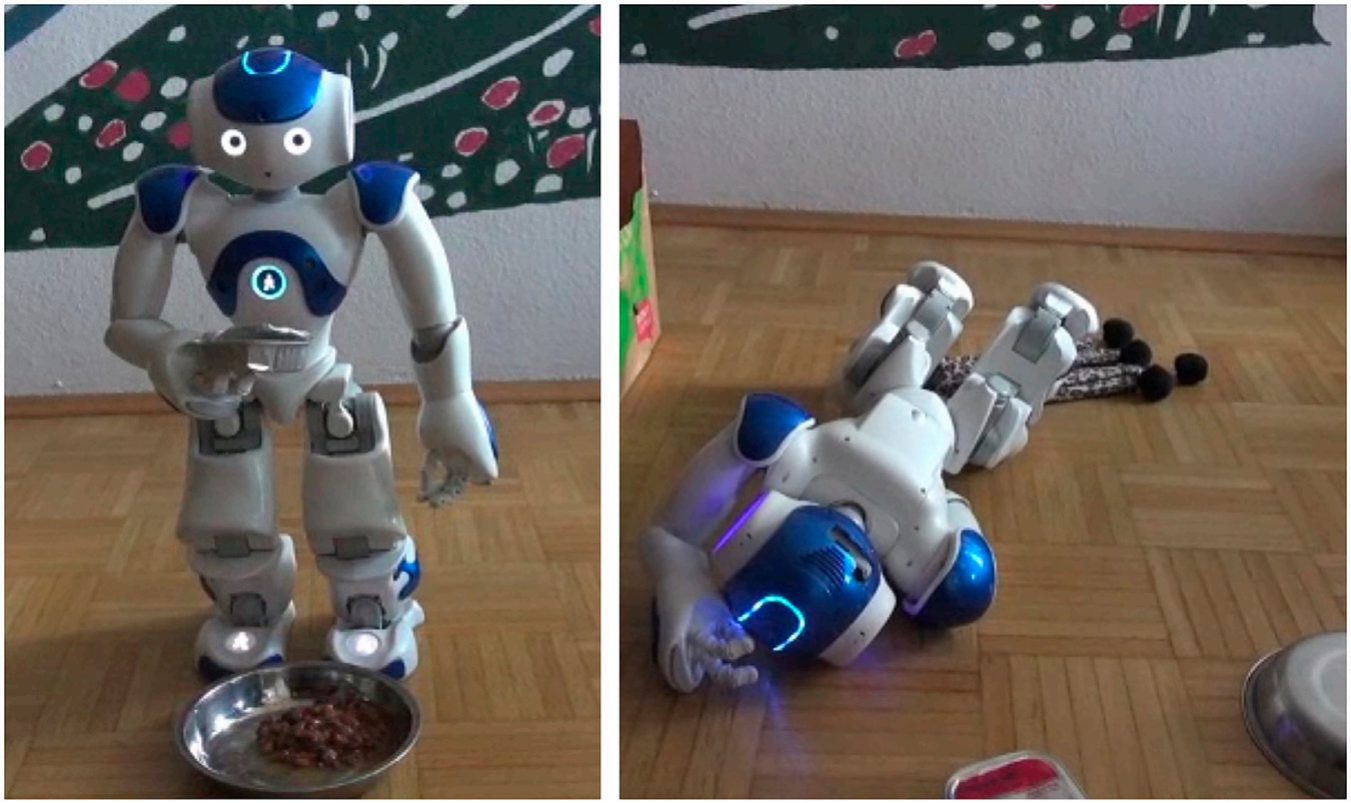
Video stills from the positive (left) and negative (right) demonstration clips.

Before the start of the safari trip, the participants were shown a video clip containing the demonstration of the robots' capability (positive or negative, see Figure 3) and several (positive or negative) customer reviews, depending on the assigned experimental condition.

#### Measures

The initial questionnaire included a manipulation check where the participants indicated their trust in the feeding robot after watching each of the manipulation stimuli, the video, and customer review (single item measure on a 7-point scale, 1 = low and 7 = high). The initial questionnaire administered before starting the safari served as a baseline measure.

Then, the safari began with a repeating daily procedure for 28 days, every day containing a decision (safari or robot check) and a resulting video (varying jungle pictures and interesting facts or an annoying car trip video and feeding results). The participants thus had to decide between *risk and reward* and *safety and no reward*. It was recorded whether the participants decided for the control call or the safari, which also allowed us to calculate the "cat death rate" after the four-week period. After each safari week, the participants filled another questionnaire with trust measurements. For these repeated trust evaluations during the study, we used the trust scale by Schaefer (Wagner, 2009), consisting of 14 items measured on a 11-point scale (0 = low and 10 = high), and averaged to a total trust score.

After the four safari weeks, the participants filled in a final questionnaire with control variables, demographic, and general questions to check for external validity (e.g., whether the participants owned a pet and how realistic they found the scenario).

### Results

#### Overview of Analyses

In the following sections, we first present manipulation checks and preliminary analyses of our data, testing the effectiveness of our manipulations (e.g., whether participants actually preferred the jungle trip as an attractive option), questions of external validity (e.g., how realistic participants perceived the scenario and the pet feeding robot), and the impact of control variables (e.g., the potential impact of owning a pet in reality on the cat death rate in our study). In general, we performed overall analyses (i.e., analyses of variance and general linear model analyses) testing the combined effects of experience (i.e., time), reputation, and capability demonstration in one model if possible. However, for reasons of clarity and comprehensibility, we report the results in three separate sections, each referring to one of the three studied influencing factors of trust (experience, reputation, and demonstration), referring to the three central dependent variables, namely trust (attitude), control calls (behavioral trust), and cat death rate.

#### Manipulation Checks and Preliminary Analyses

The manipulation checks confirmed the successful operationalization of reputation and capability demonstration. A multivariate analysis of variance with the two experimental factors reputation and capability demonstration as between subject factors and the manipulation check trust ratings as dependent measures revealed that participants in the positive reputation condition, who saw the positive reviews, provided higher trust ratings than those who saw negative reviews (M = 4.35 vs. M = 2.40, F(1,106) = 58.200, *p* < 0.001, ƞ^2^ = 0.354). Similarly, positive demonstrations resulted in higher trust ratings than negative demonstration (M = 4.00 vs. M = 2.46, F(1,106) = 25.827, *p* < 0.001, ƞ^2^ = 0.196). Furthermore, one sample *t* tests checked if the jungle trip represented an effective reward operationalization. In fact, participants' ratings confirmed that they liked the jungle videos (M = 1.97, 5-point scale, 1 = agree, 5 = disagree, T(108) = 9.46, *p* < 0.001, d = 0.906), found the jungle facts interesting (M = 1.76, T(108) = 13.729, *p* < 0.001, d = 1.315), and liked it more than the car trip (M = 1.79, T(108) = 13.542, *p* < 0.001, d = 1.297). The fact that for all three ratings, the deviations from the scale midpoint were significant, speaks for a successful manipulation of the jungle trip as an effective reward. Furthermore, the participants felt they behaved in the study just the same as they would have done in reality (1 = just the same, 7 = completely different, M = 2.01, T(108) = 9.77, *p* < 001, d = 0.936). The presented pet feeding robot was rated as moderately realistic (1 = not realistic, 7 = realistic, M = 3.81, T(109) = 1.01, n.s.). Finally, we asked if the participants owned a real pet. 42.7% of all participants answered this question positively, with cats or dogs as the most mentioned pets.

Overall, we observed a cat death rate of 58.2%, meaning out of all 110 cats only 46 survived across all conditions. Among pet owners, the cat death rate was slightly lower (51%) than among participants not having a pet (63%), but the difference in death ratios was not significant (χ^2^ (1) = 1.709, n.s.).

#### Effect of Experience

**FIGURE 4:**
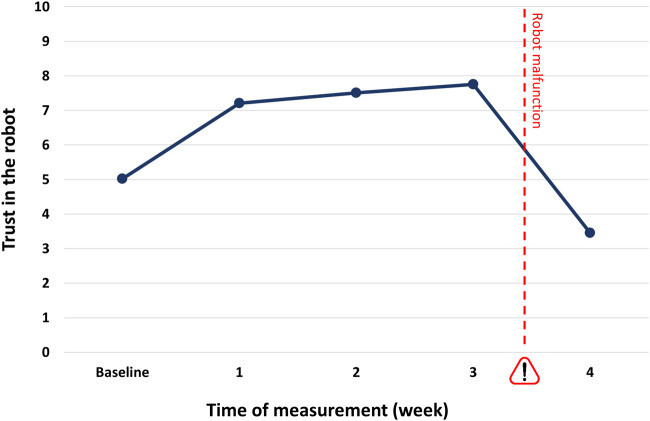
Trust ratings (range: 0–10) for baseline and four measurement points. The pet feeding robot's malfunction in the last week is indicated by the exclamation mark.

The jungle trip lasted for 28 days and included five measurement points for participants' trust in the system (baseline and after each week). This allowed us to investigate the effect of experience on trust over time. A general linear model (GLM) analysis with the five trust ratings as within-subjects factor and the two experimental factors (reputation and capability demonstration) as between-subjects factors revealed a significant main effect of experience (i.e., time) on trust (F(4,424) = 245.80, *p* < 0.001, ƞ^2^ = 0.699). Within-subjects contrasts revealed significant effects between all measurement points: While the mean trust rating for the baseline measurement was 5.0, it increased significantly to 7.2 after one week (F(1,106) = 176.30, *p* < 0.001, ƞ^2^ = 0.625). This trend continued in the following two weeks, with trust levels of 7.5 and 7.8, respectively (week 1 vs. 2: F(1,106) = 19.51, *p* < 0.001, ƞ^2^ = 0.155; week 2 vs. 3: F(1,106) = 15.538, *p* < 0.001, ƞ^2^ = 0.128). Then, ratings dropped significantly to 3.4 in week 4, reflecting the experiences with the malfunctioning robot (F(1,106) = 418.81, *p* < 0.001, ƞ^2^ = 0.798, see Figure 4).

A second general linear model (GLM) analysis explored trust on a behavioral level, that is, the performed control calls. The number of control calls for each of the four weeks was considered as within-subjects factor and the two experimental factors (reputation and capability demonstration) were considered as between-subjects factors. A significant main effect of experience (i.e., time) emerged (F(3,318) = 25.16, *p* < 0.001, ƞ^2^ = 0.19). The participants made 3.2 calls on average in the first week. Within-subjects contrasts showed that the number of calls significantly decreased in the following two weeks to 2.6 and 2.3 calls, respectively (F(1,106) = 39.98, *p* < 0.001, ƞ^2^ = 0.274; F(1,106) = 14.34, *p* < 0.001, ƞ^2^ = 0.119). Followed by a rebound to 2.6 (F(1,106) = 6.48, *p* = 0.012, ƞ^2^ = 0.058) in the final week.

#### Effect of Reputation

**FIGURE 5:**
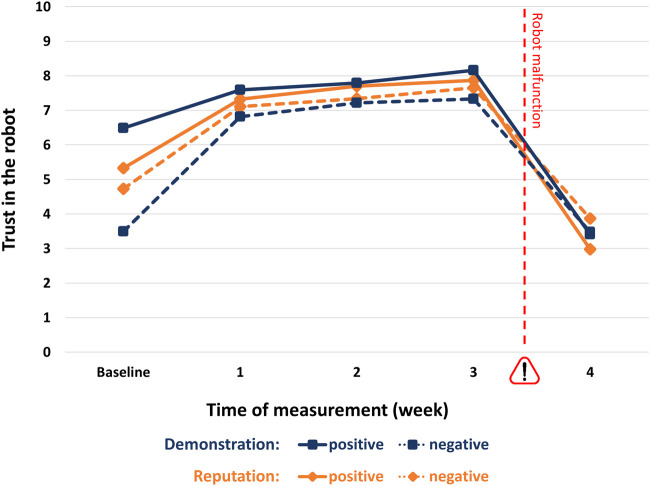
Trust ratings (range: 0–10) for positive and negative reputation and demonstration conditions.

The above described GLM analysis with the five trust ratings as within-subjects factor and the two experimental factors (reputation and capability demonstration) as between-subjects factors revealed no significant main effect of reputation (F(1,106) = 0.12, n.s.) but a significant interaction effect between reputation and experience (i.e., time) (F(4,424) = 5.55, *p* < 0.001, ƞ^2^ = 0.05). Figure 5 depicts the trust ratings (range: 0–10) for the four different experimental conditions over the course of time.

A multivariate analysis of variance with the two experimental factors (reputation and demonstration) and the trust ratings for the five points of measurements as dependent variables showed significant differences between the two reputation conditions for the baseline ratings, with initially higher trust in the positive reputation condition (positive vs. negative: M = 5.3 vs. 4.7, F(1,106) = 4.11, *p* < 0.05, ƞ^2^ = 0.037). In the then following weeks, the ratings converge with no significant differences between the reputation conditions, indicating that the effect of reputation is no longer relevant. Only for the final measurement again, a statistically significant difference emerges, however, indicating lower trust in the positive reputation condition (positive vs. negative: M = 2.98 vs. 3.87; F(1,106) = 6.14, *p* < 0.05, ƞ^2^ = 0.055).

The above described GLM analysis with control calls per week as within-subjects factor and the two experimental factors (reputation and capability demonstration) as between-subjects factors revealed no significant main effect of reputation on the number of control calls (F(1,106) = 3.84, n.s.). Also, reputation had no effect on the cat death rate (positive vs. negative reputation: 61 vs. 55%, χ(1) = 0.457, n.s.).

#### Effect of Capability Demonstration

The above described GLM analysis with the five trust ratings as within-subjects factor and the two experimental factors (reputation and capability demonstration) as between-subjects factors revealed a significant main effect of capability demonstration (F(1,106) = 20.03, *p* < 0.001, ƞ^2^ = 0.159) and also a significant interaction effect between capability demonstration and experience (i.e., time) (F(4,424) = 22.61, *p* < 0.001, ƞ^2^ = 0.176), but no significant three-way interaction between reputation, capability demonstration, and experience (F(4,424) = 1.81, n.s.).

The above described multivariate analysis of variance with the two experimental factors (reputation and demonstration) and the trust ratings for the five points of measurements as dependent variables showed significant differences between the two capability demonstrations for three of the five measures (baseline, week 1, and week 3), whereby a positive demonstration resulted in higher trust ratings than the negative demonstration. However, except of the baseline measures, the differences and effect sizes were quite small (positive vs. negative reputation: baseline: M = 6.5 vs 3.5, F(1,106) = 122.59, *p* < 0.001, ƞ^2^ = 0.536; week 1: M = 7.6 vs. 6.8, F(1,106) = 5.92, *p* = 0.017, ƞ^2^ = 0.053; week 3: M = 8.2 vs. 7.3, F(1,106) = 7.01, *p* = 0.009 , ƞ^2^ = 0.062).

The above described GLM analysis with control calls per week as within-subjects factor and the two experimental factors (reputation and capability demonstration) as between-subjects factors revealed no significant main effect of capability demonstration on the number of control calls (F(1,106) = 0.82, n. s.). Also, capability demonstration had no effect on the cat death (positive vs. negative demonstration: 64 vs. 52%, χ^2^(1) = 1.74, n.s.).

#### Interpretation of Study Findings Regarding the Development of Overtrust

**FIGURE 6:**
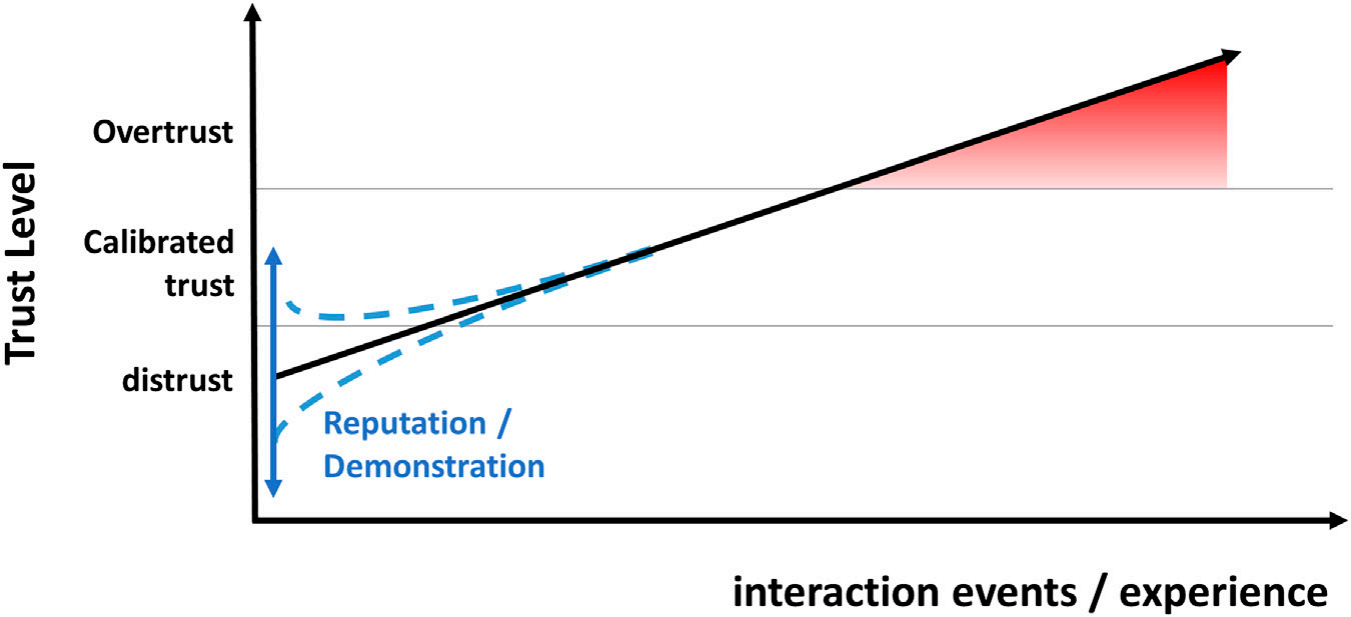
Development of trust beyond system capabilities.

A central aim of our study was to test the expected development of overtrust by simulating the typical dynamics of experience with technology over time. In line with the assumed general paradigm, repeated positive experience with the pet feeding robot leads to a continuous increase in trust and eventually overtrust on a behavioral and attitudinal level for the majority of participants. Reputation and demonstration had less influence and were primarily relevant for trust measured as a baseline, that is, before the participants could gain any personal experience themselves. Thus, in a simplified scheme, demonstration and reputation form relevant factors for the base level of trust. After this, one's positive or negative experience with the intelligent technology determines the further development of trust. If the experience is repeatedly positive, as in the first trials of our study, this results in trust even beyond the level of calibrated trust. Figure 6 illustrates this.

In general, given our findings about the primary role of subjective experience (i.e., demonstration and personal experience) compared to cognitive insight (e.g., reputation), interventions that relate to the users' subjective experience may appear more helpful than rational persuasion (e.g., "Warning, do not use the system for other purposes than intended"). In the following sections, we discuss our study findings and other cases of overtrust from a wider perspective and highlight additional psychological mechanisms that might explain user behavior, the development of overtrust. Finally, we suggest potential countermeasures and design approaches toward calibrated trust.

## General Discussion

### Instant Rewards and Lack of Falsification

A main reason for the creation of overtrust seems to be the predominance of positive short-term feedback. Initial information such as reputation or demonstration is quickly outweighed by short-term rewards and positive experiences. As long as there is no obvious reason to distrust, people follow the more comfortable way, assuming that the robot is reliable. In our study, this resulted in the participants’ decision for the safari instead of the control call. Psychologically, this is quite comprehensible. First, it is well known from consumer choice that people have a natural preference for hedonic, experiential options (here: the safari) over pragmatic options (here: the control call), especially if they can find a reason to justify their choice (e.g., Böhm and Pfister, 1996; Okada, 2005). When translating this to our scenarios, justifications for the hedonic choice may include: the robot performed well so far, why should this change, everybody would have done the same.

Second, people have a general tendency to "test" their assumptions by positivist approaches, searching for confirming information, instead of the more informative contradictory information, the so-called confirmation bias (e.g., Bye, 2012). A classical task to demonstrate this bias is the Wason card task, confronting participants with four cards showing letters or numbers (i.e., A, D, 4, and 7) and a rule about the four cards, namely "if a card has a vowel on one side, then it has an even number on the other side." Participants are then asked which card(s) they need to turn over in order to determine if the rule is true or false. The logically correct answer is to choose A and 7. A is necessary to check whether it has an even number on the other side (otherwise the rule would be falsified); 7 is necessary to check whether it has no vowel on the other side (otherwise the rule would be falsified). However, only about 4% of the participants give this correct answer. The most prominent answer is A and 4, obviously displaying a wish for confirming information. However, in order to retain reliable information about whether an assumption is true or not, one needs to search for situations in which the assumption could possibly be falsified and not situations which are compatible with existing assumption anyway. This is also proposed by Popper's scientific method of falsificationism (e.g., Percival, 2014): Instead of proposing hypotheses and then checking if they can be confirmed by evidence, Popper suggests making conjectures that can potentially be refuted. In the domain of technology this means: if you want to find out about the reliability, you have to confront technology with tasks at the expected limit of its capability.

### Inappropriate Generalization and Lack of Differentiation

Another mechanism behind overtrust could be inappropriate generalization from one successful experience to a general capability or, in other scenarios than the pet feeding robot, the lack of differentiation between situations of varying difficulty. As the example of overtrust in the Tesla autopilot showed, people generalize from positive experience in situation A that the system will be able to handle situation B as well. They seem to apply a global concept of trust toward technology similar to that of trust toward humans. Of course, even for humans, a global trust concept does not always hold true (e.g., "My wife is a fantastic driver, I trust she must be a fantastic pilot as well"). But in general, a human might detect what skills from other domains might be transferrable (e.g., "I never played badminton—but it looks a bit like tennis, let's try it with similar moves"), so that trust generalization can to some degree be adequate. For technology, it depends on whether the new situation has been defined beforehand and provides any triggers to activate helpful system skills. Even if a task seems quite "easy" to a human, a robot may not be able to solve it if its algorithms did not define any reaction for it. However, people might lack an exact concept of a technology's capabilities and limitations. If a robot can do stunning things and impress people in one domain, they may see it as a "magician," and readily believe it could do anything. Accordingly, Wagner et al. (2018) already emphasized the importance of mental modeling research and building robots that are more transparent, allowing people to fully understand how the technology will behave.

### Transfer of Social Concepts From Human–Human Interaction

As mentioned in the previous section, people tend to transfer concepts from human–human interaction (e.g., the concept of global trust) to human–robot interaction. This tendency also becomes visible in the relative effect of experience vs. reputation. A main finding of our study was that the participants' decision to trust the robot (and the cat death rate) primarily depended on their personal prior positive experience with the robot, whereas the reputation was less relevant. People may follow a rationale of "If I personally have experienced the robot to perform well so many times, it won't let me down the next time." On the contrary, others' shared experiences about the robot's performance were not crucial. Interestingly, this pattern parallels a typical and sensible behavior from human–human interaction: The reliance on personal experience for attitude formation. Even if others tell me about a person, I will build my own opinion based on my own experience. Even though others think that a person is not trustworthy, my relationship to this person can be a different one. I might have a special connection with this person and trust that he or she will never disappoint me. The same counts vice versa: others may have experienced the person as trustworthy, but I have not. Our findings suggest that people may transfer a learnt and sensible behavioral pattern from human–human interaction to human–computer interaction. In parallel to previous studies, showing that people often transfer behavioral patterns from human–human interaction (e.g., rules of courtesy, self-serving attribution biases, and group conformity) to the interaction with computers (e.g., Kiesler and Goetz, 2002; Goetz et al., 2003; Robins et al., 2004; Syrdal et al., 2007), participants behave toward the robot as if the robot had a personal relationship with them and might be more reliable for them than for others. Consequently, they disregard the valuable information they could get from others' experience reports.

### Wishful Thinking

Finally, wishful thinking may also play a role for the phenomenon of overtrust. As we know from everyday experience in many contexts, people often do not want to hear about negative aspects or potential risks, given that this would question the current comfortable way of usage. This may pertain to individual behaviors such as the risks of smoking or unhealthy nutrition but also risks on a global level such as nuclear energy, where many people do not want to hear the technology could fail. In fact, the discussion about nuclear energy could be interpreted in parallel to the partly irrational behavior as it appeared in our study: Reputation has no effect at all: In spite of the scientific and media reports about the dangers of nuclear energy, people "trust" it will never fail. Demonstration has a temporary effect: Briefly after the nuclear disasters in Chernobyl and Fukushima, governments around the world decided to ban this technology, but as the memory faded only a few years later, these decisions started to crumble as well. Experiences with the machine dominate other information: In the everyday operation of nuclear power plants worldwide, the positive experience (no direct emissions and plenty of supposedly "clean" energy) by far outweighs the knowledge about the imminent dangers, which creates a widely positive attitude and a flourishing nuclear industry.

Hence, one may question whether it is genuine trust in the technology or to some degree wishful thinking which makes many people still consider nuclear energy a safe technology. In fact, wishful thinking may be particularly pronounced, if people feel that there is no alternative to trusting the technology (e.g., a lack of convincing alternatives to nuclear energy at a large scale). Wishful thinking can also function as a way of dissonance reduction. As dissonance theory (Festinger, 1957) assumes, people strive for conformity between their attitudes, beliefs, and behaviors. If a conflict or dissonance occurs, they typically alter one of the elements. For example, if I do not want to give up smoking, I may alter my belief from "smoking is unhealthy" to "it has never been fully proven that smoking is unhealthy, actually many smokers get quite old" etc. Regarding our study scenario of trusting a pet feeding robot for being able to enjoy a safari trip, a similar mechanism could. If I do not want to change my behavior (e.g., go to the safari instead of doing the boring way to town to make a control call) I better adjust my beliefs (e.g., I trust that the robot is 100% reliable and there is no risk for my pet).

### Overtrust From a Phenomenological Perspective and Implications for Design

In the end, this leads to a quite academic discussion whether it is actually trust, altered beliefs, wishful thinking, or any similar factor which is the driving force behind overtrust and risking a technology's failure. On a phenomenological level, all these forces may affect behavior in the same way as genuine trust. This is why we consider it helpful to use the term overtrust in a wider sense for all cases in which people apply a technology beyond the limit of its capability or reliability. If we know that people are prone to the psychological mechanisms discussed above, this implies opportunities but also increased responsibilities for design. The more impressive and overwhelming the technological advancements in various domains, the more difficult it becomes for people to imagine what technology can do or cannot do, and to adequately assess a system's capabilities and limits. Designers must find ways for how a system effectively communicates its features and limits. As discussed under the notion of explainable AI (Monroe, 2018), designers have an ethical responsibility to ensure that their systems explain their strengths and weaknesses to the users and justify their suggestions and decisions in order to prevent unjustified projections and inappropriate trust. In many contexts of HCI design, using psychological mechanisms is actually helpful, for example, using metaphors or designing computer dialogs in parallel to dialogs in human–human interaction. On the other hand, design needs to foresee potential problems resulting from this transfer process and make sure that people do not transfer concepts against their own interest, for example, interpreting an autopilot in parallel to a human driver, which can easily transfer skills from one situation to others.

A central question is how to avoid overtrust and how to support calibrated trust without educating people to generally mistrust technology. Previous suggestions often described "intelligent" system reactions as a possible solution, for example, robots being able to generate information about the person's attentive state (Böhm and Pfister, 1996). However, in order to widen this perspective, we explore how to counteract overtrust by understanding its psychological foundations, including approaches that might not look like smart system behavior at all. A straightforward way to avoid the development of overtrust could be to prevent exclusively positive experience by (harmless) preprogrammed system failure at regular intervals. If, for example, your intelligent fully automatic coffee machine pours too much water into the coffee cup about every third time you press the espresso button, you probably will not trust the machine and leave the room after starting the coffee. Although the coffee tastes excellent, you would feel the machine is not reliable and you better have an eye on it. Of course, this approach of preprogrammed system failure is questionable for several reasons. It causes unneeded difficulties for the user and unneeded negative reputation for the manufacturer. Another, probably more realistic approach could be to work with implicit cues of imperfection (e.g., imperfect grammar in dialog systems), reminding the user that the technology does not work as accurately as the user may assume. From a psychological perspective, such little quirks may even make it appear more human and likable. As revealed in previous research on peoples' relationships with their technical products, a little friction in system interaction is even interpreted as a part of a positive relationship and forgiving statements such as "It [the smartphone] behaves like a modest, loyal servant also be a bit funny—sometimes a program doesn't work properly—it's not the perfect support. But little quirks also make it more likable, more humane" (Chris, cited after Diefenbach and Hassenzahl, 2019, p. 11).

## Limitations and Future Research

At least four basic limitations need to be considered for the interpretation of our findings. The first and most general limitation refers to the nonrepresentative sample of participants, that is, rather young people within a limited age range, most of them having an academic background. Although there is no obvious indication that overtrust should be less frequent among older or nonacademic samples, future studies should include more diverse samples of participants.

The second aspect refers to the study's external validity and quantitative focus. Participants' decision to trust the robot or not could realistically affect their emotional experience (i.e., seeing jungle pictures and interesting facts when trusting the robot or an annoying car trip video and feeding results when not trusting the robot) but the risk related to trusting the robot (i.e., the cat dies) was only fictional. Hence, one could question whether the participants would have made the same choice if their real pet's life was in danger. Also, our study was focused on quantitative measures of trust and there was no qualitative assessment of the participants' subjective feelings and how they experienced the scenario. It should be noted, however, that the main aim of our research was the exploration of the assumed paradigm of overtrust and possible additional influencing factors. Even though the general trust rates might have been slightly different if studied in a real-life setting, there is no obvious reason to assume that this would have changed the relative effect of the influencing factors experience, reputation, and demonstration. Future studies should include field studies and complement quantitative accounts with qualitative approaches.

Third, our study was limited to three influencing factors of overtrust (personal experience, reputation, and demonstration) which we identified as dominant in the literature and our pre-study. Hence, while our model provides a valid starting point and framework for the study of overtrust, future research should extend this by an exploration of further influencing factors such as the different social and psychological mechanisms discussed above. Integrating such factors in future research will provide a more holistic picture of the phenomenon of overtrust, its consequences, and potential interventions.

Fourth, one may argue that the course of robot experience our study design provided (i.e., the robot performs well for repeated times and then suddenly fails) was not very realistic, especially given that failures are in themselves rather unlikely. One might even argue that our study design was "unfair" since everybody would trust a machine that has proven so many times. However, even if rarely, the same pattern may occur in real-life scenarios: the cases in which technology fails are rare and experienced only by single users. As a consequence, the most common experience (e.g., watching many people on YouTube doing funny things and taking their hands off the wheel while using the Tesla autopilot and no accident happens) does not reflect the associated risk of blindly trusting the technology. The same happens in other situations without technology being involved, for example, skiing in an avalanche risk area without any problems for many times and then getting killed one day. Above all, these repeated positive experiences make people develop inappropriate trust in a technology or situation and this is what we wanted to simulate. In sum, we created a highly artificial scenario with high internal validity, however, connected to limitations regarding external validity.

In addition to these specific limitations, future work could also further explore the connections to other psychological concepts listed in the discussion section such as inappropriate generalization or cognitive dissonance.

## Conclusion

As shown by the discussion above, the development of inappropriate trust in intelligent systems has to be seen not as the exception but as the rule. This presents a serious problem when it comes to sensitive domains in which lives or personal well-being might be at stake. The presented case study and psychological analysis make the underlying mechanisms comprehensible, yet they do not deliver any obvious general solutions. The challenge to design and develop technologies in such a way that they prompt an adequate or calibrated level of trust will remain one of the most pressing ones, as long as we are not in a position to develop systems which justify the great amount of trust they are met with by working perfectly.

## Data Availability

The data is available under https://doi.org/10.5282/ubm/data.196.

## Footnotes

1
https://en.wikipedia.org/wiki/Diffusion_of_responsibility.
